# Implementation of Nutritional Strategies Decreases Postnatal Growth Restriction in Preterm Infants

**DOI:** 10.1371/journal.pone.0051166

**Published:** 2012-12-05

**Authors:** Paola Roggero, Maria L. Giannì, Anna Orsi, Orsola Amato, Pasqua Piemontese, Nadia Liotto, Laura Morlacchi, Francesca Taroni, Elisa Garavaglia, Beatrice Bracco, Massimo Agosti, Fabio Mosca

**Affiliations:** 1 Neonatal Intensive Care Unit (NICU), Department of Clinical Science and Comunity Health, Fondazione IRCCS “Ca' Granda” Ospedale Maggiore Policlinico, University of Milan, Milan, Italy; 2 Maternal and Child Health Department, Del Ponte Hospital, A.O. Di Circolo Fondazione Macchi, Varese, Italy; Emory University School of Medicine, United States of America

## Abstract

**Background:**

Prevention of postnatal growth restriction of very preterm infants still represents a challenge for neonatologists. As standard feeding regimens have proven to be inadequate. Improved feeding strategies are needed to promote growth. Aim of the present study was to evaluate whether a set of nutritional strategies could limit the postnatal growth restriction of a cohort of preterm infants.

**Methodology/Principal Findings:**

We performed a prospective non randomized interventional cohort study. Growth and body composition were assessed in 102 very low birth weight infants after the introduction of a set of nutritional practice changes. 69 very low birth weight infants who had received nutrition according to the standard nutritional feeding strategy served as a historical control group. Weight was assessed daily, length and head circumference weekly. Body composition at term corrected age was assessed using an air displacement plethysmography system. The cumulative parenteral energy and protein intakes during the first 7 days of life were higher in the intervention group than in the historical group (530±81 vs 300±93 kcal/kg, p<0.001 and 21±2.9 vs 15±3.2 g/kg, p<0.01). During weaning from parenteral nutrition, the intervention group received higher parental/enteral energy and protein intakes than the historical control group (1380±58 vs 1090±70 kcal/kg; 52.6±7 vs 42.3±10 g/kg, p<0.01). Enteral energy (kcal/kg/d) and protein (g/kg/d) intakes in the intervention group were higher than in the historical group (130±11 vs 100±13; 3.5±0.5 vs 2.2±0.6, p<0.01). The negative changes in z score from birth to discharge for weight and head circumference were significantly lower in the intervention group as compared to the historical group. No difference in fat mass percentage between the intervention and the historical groups was found.

**Conclusions:**

The optimization and the individualization of nutritional intervention promote postnatal growth of preterm infants without any effect on percentage of fat mass.

## Introduction

The prevention of postnatal growth restriction of very preterm infants still represents a challenge for neonatologists [1]. As standard feeding regimens have proven to be inadequate, improved feeding strategies are needed to promote growth. Indeed, the achievement of an adequate growth has been associated with positive neurodevelopment outcomes [2], [3]. In addition, preterm infants have been reported to show higher adiposity at term corrected age that could be due to insufficient protein intake during hospital stay [4], [5].

The prevention of severe nutrient deficits during hospital stay may be achieved through the implementation of the knowledge of macronutrients/micronutrients needs, the optimization of nutritional policies and the individualization of the nutritional intervention [6]. Senterre et al. [7], [8] have recently demonstrated that the nutritional supply optimized to reflect the more recent recommendations can limit the postnatal growth restriction. Consistently with these findings, Rochow et al. [9] further showed that the daily adjustment of protein and energy intakes according to the individual growth trajectory improves growth of preterm infants without any modification of body composition. Costa-Orvay et al. [10] found that the weight increase was higher in infants receiving higher enteral protein intakes than in infants receiving standard intakes. In addition, the weight increase reflected a greater accretion in fat free mass.

The aim of the present study was to evaluate whether a set of nutritional strategies, directed to optimize and individualize the nutritional regimen according to the more recent recommendations [11], [12] could limit the postnatal growth restriction and the development of adiposity at term corrected age in a cohort of preterm infants. The hypothesis to be tested is that preterm infants receiving an optimized and individualized nutritional regimen would show better growth, both in quantity and in quality, during hospital stay than a historical control group of preterm infants who had received nutrition according to the standard nutritional feeding strategy.

**Table 1:** Macronutrients intakes during the first week of life in the intervention (IG) and historical (HG) groups.

DOL*	Glucose (g/kg)		Proteins (g/kg)		Lipids (g/kg)		Water (ml/kg)	
	IG	HG	IG	HG	IG	HG	IG	HG
1	10	8	2.5	1.5	0.75	0	70–100	60–100
2	10	9	3	2	1.5	0.75	70–110	70–110
3	12	11	3	2.5	1.5	1.5	80–120	80–120
4	14	13	3.5	3	2	1.5	100–140	100–140
5	14	14	3.5	3	2.5	2	120–150	120–160
6	14	14	4	3.5	3	2.5	140–170	140–170
7	14	14	4	3.5	3	3	150–180	150–180

* Day of life.

## Materials and Methods

This study was approved by the Ethics Committee of the Fondazione IRCCS “Ca Granda” Ospedale Maggiore Policlinico.

**Table 2:** Basic characteristic of the infants studied.

	Intervention Group	Historical group	p value
Infants n	102	69	
Males n	53 (52)	32 (46.4)	0.5
GA weeks	30.05 (2.1)	30.08 (2.3)	0.9
Birth Weight z score	−0.8 (0.8)	−1.07 (0.9)	0.07
Birth Length z score	−0.7 (1.1)	−1.0 (1.3)	0.7
Birth Head Circumference z score	−0.68 (1)	−0.78 (1)	0.6
SGA n	34 (33.3)	25 (36)	0.6
ELBW n	24 (23.5)	22 (31.9)	0.6
CLD n	6 (5.9)	7 (10.1)	0.2
IVH>2°n	3 (2.9)	4 (5.7)	0.4
Sepsis n	18 (17.6)	14 (20.3)	0.7
ROP>2°n	1 (0.9)	1 (1.4)	0.9

Data are expressed as mean (SD) or number of observations (percentage).

GA: gestational age; ELBW: extremely low birth weight infants.

### Subjects

We performed a prospective non randomized interventional cohort study in infants with a birth weight <1500 g. Exclusion criteria were: infants who died during hospital stay, infants with congenital anomalies and/or cardiac and/or gastrointestinal diseases and infants who were early transferred to other units.

**Figure 1:**
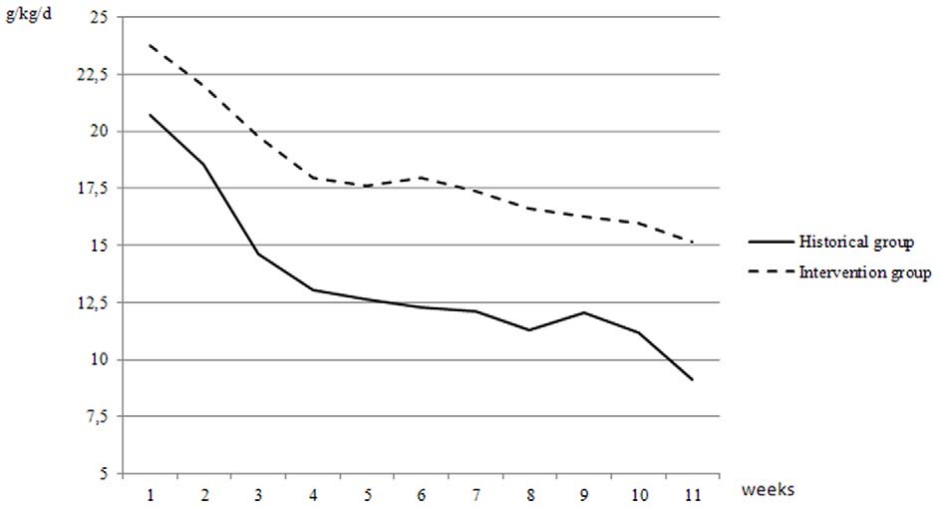
Weight velocity (g/kg/day) in the intervention and in the historical groups.

The intervention group included infants admitted to our Institution during the period from January 2009 to December 2010, after the introduction of a set of nutritional practice changes to our standard feeding strategy. These infants were compared with a historical control group that comprised infants admitted from January 2005 to December 2006, who had received nutrition according to the standard nutritional feeding strategy.

Neonatal characteristics (gestational age, being appropriate or small for gestational age, birth weight, length, and head circumference); the occurrence of sepsis, chronic lung disease (CLD), retinopathy of prematurity of stage 3 or higher (ROP) and intraventricular hemorrhage of stage 3 or higher (IVH) were recorded prospectively. Gestational age was based on the last menstrual period and first-trimester ultrasonogram. Infants with birth weight >10th or <10th percentile for gestational age, according to the Fenton's growth chart [13], were classified as appropriate for gestational age (AGA) or small for gestational age (SGA), respectively. Sepsis was defined by the presence of a positive blood culture. Chronic lung disease was defined by use of supplemental oxygen at 36 weeks' postconceptional age. Corrected age was calculated from the chronologic age adjusting for gestational age.

**Table 3:** Anthropometric parameters and gestational age at discharge.

	Historical Group	Intervention Group	P
z-score for weight	−2.2 (0.7)	−1.7 (0.7)	0.001
z-score for length	−2.2 (1.3)	−1.8 (1.17)	0.1
z-score for HC*	−1.4 (0.8)	−0.9 (0.6)	0.002
Gestational age (weeks)	38.8 (2.6)	37.5 (2.4)	0.001

* HC: head circumference.

Data are expressed as mean (SD).

### Nutritional regimens

#### Intervention group

The nutritional intervention was focused on parenteral nutrition, the weaning from parenteral nutrition and enteral nutrition.

**Figure 2:**
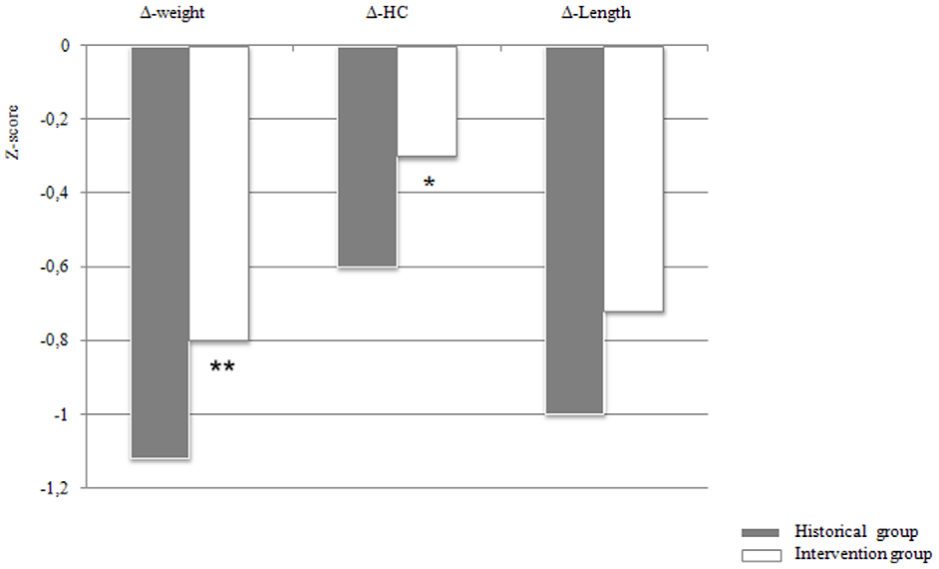
Growth z score changes from birth to discharge in the intervention and historical groups * p = 0.04; ** p = 0.01.

**Figure 3:**
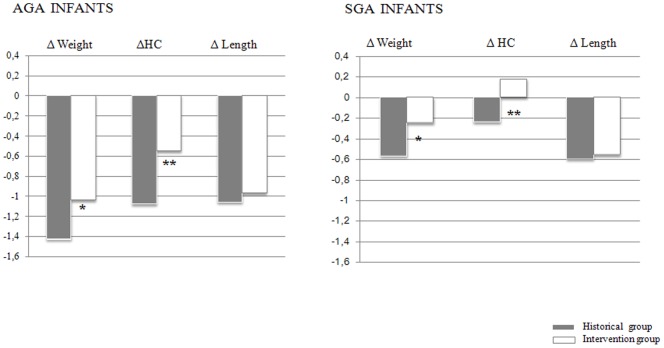
Growth z score changes from birth to discharge in AGA (*p = 0.008; **p = 0.003) and SGA infants (*p = 0.04; **p = 0.02).

The parenteral solutions were prepared by the hospital pharmacy according to medical prescription. They supplied a minimum of 57 kcal/kg/day with 2.5 g/kg of proteins on the first day of life, up to 90–100 kcal/kg/day and 4 g/kg/day of proteins within the first week (table 1).

The weaning from parenteral nutrition was scheduled in order to obtain a weight velocity >15 g/kg/day. To accomplish this goal, the macronutrients, especially proteins, were reduced gradually according to weight velocity.

Enteral feeding was initiated within 24 hours of postnatal life using breast milk or preterm formula when breast milk was absent (energy: 83 kcal/100 ml; carbohydrates: 8.4 g/100 ml; proteins: 2.9 g/100 ml; fat: 4.1 g/100 ml). When the infants tolerated an enteral intake ≥100 ml/kg, individually tailored fortification of breast milk was started. Enteral protein and energy intakes were adjusted on a day-to-day basis in order to maintain a weight velocity >15 g/kg/day. With regard to energy provision, Duocal (Nutricia, Germany) and MCT oil (Nestle, Switzerland) were added to reach a mean of energy intake of 120 kcal/kg/day. Supplementation with Duocal ranged from 1 g up to 5 g in 100 ml; MCT oil was added starting from 1 ml to 3 ml in 100 ml. With regard to protein provision, FM 85 (Nestle, Switzerland) and Protifar (Nutricia, Germany) were added to reach a mean of protein intake of 3.5 g/kg. Five grams of FM 85 were added in 100 ml; supplementation with Protifar ranged from 1 to 2.5 g in 100 ml.

#### Historical control group

Parenteral nutrition supplied a minimum of 38 kcal/kg and 1.5 g/kg of protein on the first day up to 90 kcal/kg/day and 3.5 g/kg/day of protein within the first week (table 1). During the weaning from parenteral nutrition the macronutrients administered parenterally were calculated from the difference between the global scheduled macronutrients intake and the macronutrients given by enteral route. Enteral feeding was initiated within 24–48 hours of postnatal life with breast milk or, when breast milk was absent, preterm formula. When the infants tolerated an enteral intake ≥100 ml/kg, standard fortification of breast milk (FM 85 Nestle, Switzerland; 5 g/100 ml of breast milk) was started.

### Nutritional and Growth data

#### Nutrition

In order to facilitate the nutritional prescriptions and to promote the adherence to the new nutritional feeding program an electronic medical record system has been introduced. The attending neonatologist was in charge for monitoring the compliance to the new feeding program.

Energy and protein parenteral and enteral intakes were calculated daily from the patients' computerized medical charts from birth to discharge. Cumulative nutritional intakes were calculated as the sum of the daily intakes.

#### Growth and body composition measurements

Growth and body composition measurements were assessed by three medical investigators. Body weight, length and head circumference were measured according to standard procedures. Weight was assessed daily; length and head circumference were assessed weekly. Subject mass was measured on an electronic scale accurate to the nearest 0.1 g and body length was measured to the nearest 1 mm on a Harpenden neonatometer (Holtain Ltd, UK). Head circumference was measured to the nearest 1 mm with non-stretch measuring tape. Growth z-scores were calculated at birth and term using the infant z-scores based on the Fenton preterm infant growth chart [13].

Body composition was assessed at term corrected age using an air displacement plethysmography system (PEA POD Infant Body Composition System, COSMED-USA). A detailed description of the PEA POD's physical design, operating principles, validation and measurement procedures is provided elsewhere [14], [15]. The PEA POD assesses fat mass and fat free mass by direct measurements of body mass and volume and the application of classic densitometric principles. Infants were measured in the PEA POD naked. Each PEA POD test took about 3 min to complete. Subject volume was measured in an enclosed chamber by applying gas laws that relate pressure changes to volumes of air in the chamber. Body density was then computed from the measured body mass and volume, and inserted into a standard formula for estimating the percentage of total body fat mass according to a 2-compartment model. The intra-observer coefficient of variation for the percentage of fat mass estimates was 0.3%.

Weight velocity was assessed using the following formula [1000× ln (Wn/W1)]/(Dn-D1) [W =  weight in grams; D = day; 1 = beginning of the time interval n =  the end of the time interval] [16] Changes in z score were calculated by subtracting z score at discharge from that at birth.

### Statistical analysis

Values were given as means with standard deviations or number of observations (percentage).

Differences between groups in measurements of growth parameters, fat mass and energy and protein intakes were assessed by Student's t-test analysis. X^2^ test was used for comparisons between discrete variables. Statistical significance was set at a = 0.05 level. All statistical analyses were performed using SPSS (SPSS, version 12, SPSS Inc., Chicago, IL).

## Results

One hundred seventy-one infants were included in the study. The basic characteristics of the infants enrolled in the study both in the historical and in the intervention groups are shown in table 2. No difference in the anthropometric parameters at birth and in the occurrence of CLD, IVH, sepsis and ROP between the two groups was found.

### Nutrition

The cumulative parenteral energy and protein intakes during the first 7 days of life were significantly higher in the intervention group than in the historical group (530±81 vs 300±93 kcal/kg, p<0.001 and 21±2.9 vs 15±3.2 g/kg, p<0.01). The daily average parenteral energy and protein intakes for the first week were 75±10 vs 43±12 kcal/kg and 3.0±0.4 vs 2.2±0.4 g/kg in the intervention and in the historical group, respectively.

The mean duration (d) of parenteral nutrition was similar in the historical and in the intervention groups (20.6±13 vs 20.3±9, respectively).

During the weaning from parenteral nutrition, 55% of the cumulative energy intake and 60% of the cumulative protein intake were provided by parenteral nutrition in the intervention group, whereas parenteral nutrition provided 36% of the cumulative energy intake and 50% of the cumulative protein intake in the historical group. In detail, the intervention group received significantly higher parenteral/enteral energy and protein intakes than the historical control group (1380±58 vs 1090±70 kcal/kg; 52.6±7 vs 42.3±10 g/kg, p<0.01).

After the withdrawal of parenteral nutrition, the mean enteral energy (kcal/kg/d) and protein (g/kg/d) intakes were higher in the intervention group than in the historical group (130±11 vs 100±13, p<0.01; 3.5±0.5 vs 2.2±0.6). The historical and the intervention groups required 30.7±13 and 27.5±13 days to achieve full enteral feeding, respectively (p = 0.13).

The percentage of infants that were breast fed, formula fed and mixed fed were similar in the historical and in the intervention groups (15 vs 18; 28 vs 26; 57 vs 56, respectively).

### Growth

Weight velocity in both groups is shown in figure 1. Infants in the intervention group showed a higher weight velocity than infants in the historical group (p = 0.001) throughout the study.

Anthropometric parameters and gestational age at discharge are reported in table 3. Gestational age at discharge in the historical group was significantly higher than in the intervention group. Z-scores for weight and head circumference were significantly lower in the historical group than in the intervention group.

The changes in z score from birth to discharge for weight, length and head circumference in infants of both groups are reported in figure 2. The negative changes in z score from birth to discharge for weight and head circumference were significantly lower in the intervention group as compared to the historical group. The same results have been found when considering the infants according to intrauterine growth pattern (figure 3).

Considering the AGA infants, the proportion of infants discharged with a weight less than −2 SD was lower in the intervention group as compared to the historical group (30% v 13%, p = 0.04). With regard to SGA infants, no significant difference among groups in the proportion of infants discharged with a weight less than −2 SD was detected.

### Body composition

With regard to body composition we did not find any difference in fat mass percentage between the intervention and the historical group, respectively (15.8±4 vs 16.2±3.9; p = 0.2).

## Discussion

In the present study we demonstrated that the introduction of a set of nutritional strategies, directed to optimize and individualize the nutritional regimen according to the most recent recommendations, led to the partial limitation of the postnatal growth restriction in a cohort of preterm infants. Indeed, the infants receiving the new set of nutritional strategies showed a higher weight velocity during both parenteral and enteral nutrition. As a result, the negative changes in weight and head circumference z scores from birth to discharge were significantly lower in the intervention group as compared to the historical group regardless of intrauterine growth pattern.

The infants in the intervention group were discharged at a significantly lower gestational age than the infants in the historical group as they succeeded in reaching an acceptable weight gain in a shorter length of time. The prevalence of comorbidities that could have negatively affected growth was similar in the intervention group as compared to the historical group. With regard to AGA infants, the percentage of infants discharged with a weight below the −2 standard deviations was significantly lower in the intervention group than in the historical one. With regard to infants born SGA, we actually failed to find a reduction in the percentage of infants discharged with a weight below the −2 standard deviations, although the SGA infants showed a better weight velocity and a less negative changes in weight and head circumference z scores from birth to discharge. The absence of catch up growth can be partially explained by the fact that the growth constraint experienced during the intrauterine life may delay the occurrence of recovery of growth in the SGA infants of the present study. No difference in body composition between the intervention and the historical group were found.

These findings are partially consistent with those reported by Senterre T et al. [7]. Indeed, the authors found that the optimization of nutritional policies, based on the most recent recommendations, during parenteral and enteral nutrition resulted in improved growth and limitation of postnatal growth restriction of a cohort of 102 preterm infants. In contrast to our findings the authors also reported that 20% of the infants born SGA were discharged with a weight higher than −2 standard deviations, suggesting that catch up growth can be achieved in every subgroup of infants.

Rochow N. et al. [9] recently investigated the effect of the introduction of a new set of nutritional policies on postnatal growth and body composition of 123 preterm infants in comparison to a control group that had received nutrition according to the prevailing nutritional standard. The authors reported an improved growth with regard to weight and head circumference at 36 weeks of corrected age in the intervention group without any modification of body composition assessed by DEXA. These findings confirm our results, with regard also to the absence of any effect of the implementation of nutritional strategies on body composition.

Several studies have demonstrated that high energy and protein intakes beneficially affect ponderal and head circumference growth in addition to fat free body mass accretion. Almost 3.5 g/kg/body weight of amino acids are needed to retain 350 mg of nitrogen that enables to achieve a growth of 17 g/kg/day. Protein and energy intakes need to be considered concomitantly. Indeed, proteins are oxidized instead of being used for tissue accretion when energy is lacking, whereas when a high energy diet is administered without increasing the protein intake, a disproportionate deposition of body fat mass takes place [17], [18]. Cooke et al [19] conducted a randomized controlled trial reporting a direct relationship between head growth at 36 weeks of postconceptional age and increased energy and protein intakes (r = 0.44 and r = 0.34, respectively). Miller et al [20] demonstrated that failure to thrive in preterm infants fed human milk was lower when human milk was fortified with high protein content. Costa-Orvay et al. [10] investigated the effect of high protein and high energy delivered during full enteral feeding on growth and weight gain composition. The authors demonstrated greater weight gains in preterm infants receiving high protein and energy intakes. The infants receiving high protein and energy intakes showed significantly (p = 0.002) greater weight gains than preterm infants receiving standard protein and energy intakes. The greater weight gains reflected a greater increase in fat free mass assessed by means of total body electrical impedance analysis. The findings of this study further underline the importance of the protein-energy ratio in achieving an appropriate increase in weight and fat free mass.

The weakness of the study is that it is not a randomized controlled trial. In addition, it has to be taken into account that the intervention group was compared with a control group from 4 years prior to the intervention. However, this interval time was required in order to have all the hospital staff trained for the new nutritional management and to investigate the feasibility of these new set of nutritional practices.

The strength of the study is that the nutritional intervention was based on the weight velocity of each single infant. Indeed, the use of neonatal growth charts has been reported to present some limitations in clinical practice [21].

The present study demonstrated that the optimization and the individualization of nutritional intervention, using weight velocity as a “coach” for the weaning from parenteral nutrition and for the adjustment of enteral nutrients administration, promote postnatal growth of preterm infants without any effect on body composition.
